# Solvent-Induced Formation of Novel Ni(II) Complexes Derived from Bis-Thiosemicarbazone Ligand: An Insight from Experimental and Theoretical Investigations

**DOI:** 10.3390/ijms22105337

**Published:** 2021-05-19

**Authors:** Ghodrat Mahmoudi, Maria G. Babashkina, Waldemar Maniukiewicz, Farhad Akbari Afkhami, Bharath Babu Nunna, Fedor I. Zubkov, Aleksandra L. Ptaszek, Dariusz W. Szczepanik, Mariusz P. Mitoraj, Damir A. Safin

**Affiliations:** 1 Department of Chemistry, Faculty of Science, University of Maragheh, Maragheh P.O. Box 55181-83111, Iran; 2 Independent Researcher, Respubliki Str. 14, 625003 Tyumen, Russia; maria.babashkina@gmail.com; 3 Institute of General and Ecological Chemistry, Lodz University of Technology, Żeromskiego 116, 90-924 Łódź, Poland; 4 Department of Chemistry, The University of Alabama, Box 870336, 250 Hackberry Lane, Tuscaloosa, AL 35487, USA; f.a.afkhami@gmail.com; 5 Department of Mechanical and Industrial Engineering, New Jersey Institute of Technology, University Heights, Newark, NJ 07102, USA; bn63@njit.edu; 6 Department of Medicine, Division of Engineering in Medicine, Brigham and Women’s Hospital, Harvard Medical School, Harvard University, Cambridge, MA 02139, USA; 7 Organic Chemistry Department, Faculty of Science, Peoples’ Friendship University of Russia (RUDN University), Miklukho-Maklaya Str. 6, 117198 Moscow, Russia; fzubkov@sci.pfu.edu.ru; 8 Department of Theoretical Chemistry, Faculty of Chemistry, Jagiellonian University, Gronostajowa 2, 30-387 Cracow, Poland; aleksandra.ptaszek6@gmail.com (A.L.P.); dszczpnk@gmail.com (D.W.S.); 9 Institute of Chemistry, University of Tyumen, Volodarskogo Str. 6, 625003 Tyumen, Russia; 10 Innovation Center for Chemical and Pharmaceutical Technologies, Ural Federal University Named after the First President of Russia B.N. Eltsin, Mira Str. 19, 620002 Ekaterinburg, Russia; 11 Kurgan State University, Sovetskaya Str. 63/4, 640020 Tyumen, Russia

**Keywords:** Ni(II) complexes, intermolecular C–H∙∙∙Ni interactions, σ-metalloaromaticity

## Abstract

In this work, we report solvent-induced complexation properties of a new N_2_S_2_ tetradentate bis-thiosemicarbazone ligand (**H_2_L^I^**), prepared by the condensation of 4-phenylthiosemicarbazide with bis-aldehyde, namely 2,2’-(ethane-1,2-diylbis(oxy)dibenzaldehyde, towards nickel(II). Using ethanol as a reaction medium allowed the isolation of a discrete mononuclear homoleptic complex **[NiL^I^]** (**1**), for which its crystal structure contains three independent molecules, namely **1-I**, **1-II,** and **1-III**, in the asymmetric unit. The doubly deprotonated ligand **L^I^** in the structure of **1** is coordinated in a cis-manner through the azomethine nitrogen atoms and the thiocarbonyl sulfur atoms. The coordination geometry around metal centers in all the three crystallographically independent molecules of **1** is best described as the seesaw structure. Interestingly, using methanol as a reaction medium in the same synthesis allowed for the isolation of a discrete mononuclear homoleptic complex **[Ni(L^II^)_2_]** (**2**), where **L^II^** is a monodeprotonated ligand 2-(2-(2-(2-(dimethoxymethyl)phenoxy)ethoxy)benzylidene)-N-phenylhydrazine-1-carbothioamide (**HL^II^**). The ligand **L^II^** was formed in situ from the reaction of **L^I^** with methanol upon coordination to the metal center under synthetic conditions. In the structure of **2**, two ligands **L^II^** are coordinated in a trans-manner through the azomethine nitrogen atom and the thiocarbonyl sulfur atom, also yielding a seesaw coordination geometry around the metal center. The charge and energy decomposition scheme ETS-NOCV allows for the conclusion that both structures are stabilized by a bunch of London dispersion-driven intermolecular interactions, including predominantly N–H∙∙∙S and N–H∙∙∙O hydrogen bonds in **1** and **2**, respectively; they are further augmented by less typical C–H∙∙∙X (where X = S, N, O, π), CH∙∙∙HC, π∙∙∙π stacking and the most striking, attractive long-range intermolecular C–H∙∙∙Ni preagostic interactions. The latter are found to be determined by both stabilizing Coulomb forces and an exchange-correlation contribution as revealed by the IQA energy decomposition scheme. Interestingly, the analogous long-range C–H∙∙∙S interactions are characterized by a repulsive Coulomb contribution and the prevailing attractive exchange-correlation constituent. The electron density of the delocalized bonds (EDDB) method shows that the nickel(II) atom shares only ~0.8|e| due to the σ-conjugation with the adjacent in-plane atoms, demonstrating a very weak σ-metalloaromatic character.

## 1. Introduction

The progress in the coordination chemistry of transition metals is still a compelling and experimentally demanding frontier in modern inorganic chemistry. Every year, we observe the emergence of scientific reports on the synthesis of new complexes with unexpected bonding modes, structures, and properties.

Among a variety of different ligands, which are actively used in the coordination chemistry, (thio)semicarbazones seem to be one of the most widely utilized ligands. (Thio)semicarbazones were first reported in the late 1800s to early 1900s [1] and possessed remarkable complexation properties towards a great variety of metal ions. These compounds comprise a separate family of the so-called Schiff bases, and are readily obtained through the condensation reaction of (thio)semicarbazides with aldehydes or ketones. Using precursors with two or more aldehyde or ketone functions allows us to obtain polyfunctional (thio)semicarbazones. Thus, ease of synthesis as well as pronounced complexation properties are of particular interest for a wide application of these types of compounds for smart design of different structures of interest. Generally, (thio)semicarbazone moieties, and more precisely their anionic forms, are N,O/S bidentate ligands, yielding a five-membered chelate metallocycles upon coordination to a metal ion. However, the incorporation of different additional donor functions, e.g., pyridine derivatives, can facilitate a tridentate (polydentate) coordination mode [2].

Apart from their great importance as building units in the coordination chemistry, (thio)semicarbazones as well as their metallocomplexes have actively been studied over a number of years because of their diverse biological activity and, thus, are a focus in biomedicine [2,3,4,5,6,7,8,9,10].

Some time ago, we also directed our attention toward closely related hiosemicarbazides. Particularly, we were interested in a family of (thio)phosphorylated thiosemicarbazides, including bifunctional derivatives, as potential polydentate ligands [11,12,13,14]. The reported compounds were readily obtained by the addition reaction of the corresponding hydrazine derivatives to (thio)phosphorylated isothiocyanates. Furthermore, a dramatic influence of the solvent nature was revealed for the formation of the final product [14].

**Scheme 1:**
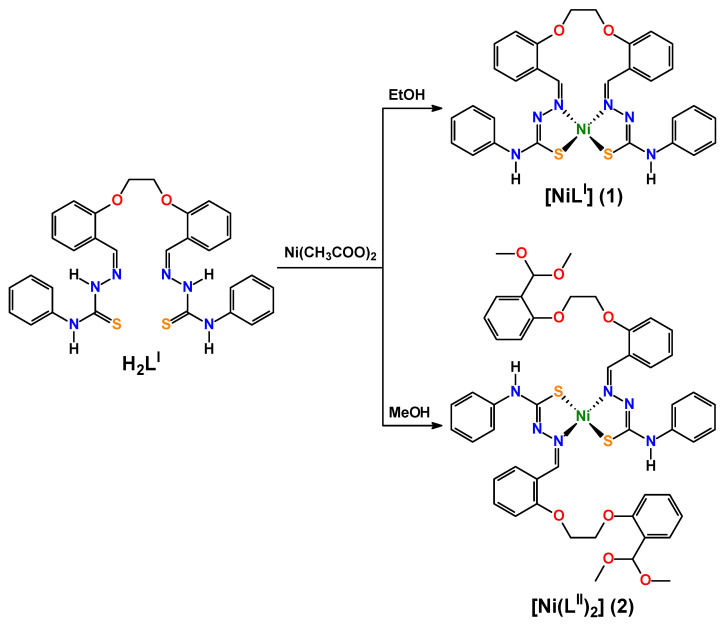
Synthesis of complexes **1** and **2**.

A wide diversity of applications thus prompted the present study in which we report the solvent-induced synthesis and the molecular and supramolecular structures of two nickel(II) complexes derived from the bis-thiosemicarbazone ligand **H_2_L^I^**, obtained by the condensation of 2,2’-(ethane-1,2-diylbis(oxy)dibenzaldehyde with 4-phenylthiosemicarbazide (Scheme 1). It should be noted, that, to the best of our knowledge, neither the crystal structure of **H_2_L^I^** nor its metallocomplexes are known so far. Thus, the chemistry of **H_2_L^I^** is of particular interest. Importantly, extensive theoretical studies are performed to identify physical factors, which contribute to the stability of the reported metal-based supramolecular architectures.

## 2. Results and discussion

A one-pot reaction of equimolar amounts of Ni(CH_3_COO)_2_·2H_2_O with **H_2_L^I^** in EtOH at 60 °C in a branched tube apparatus leads to a discrete mononuclear homoleptic complex **[NiL^I^]** (**1**) (Scheme 1). Notably, using methanol as a reaction medium in the same synthesis leads to a discrete mononuclear homoleptic complex **[Ni(L^II^)_2_]** (**2**) (Scheme 1), where **L^II^** is a monodeprotonated ligand 2-(2-(2-(2-(dimethoxymethyl)phenoxy)ethoxy)benzylidene)-N-phenylhydrazine-1-carbothioamide (**HL^II^**). The ligand **L^II^** was formed in situ from the reaction of **L^I^** with MeOH upon coordination to the metal center under synthetic conditions. Thus, the reaction of Ni(CH_3_COO)_2_·2H_2_O with **H_2_L^I^** is solvent sensitive. Both compounds were isolated as crystalline air-stable solids with good yields.

**Figure 1:**
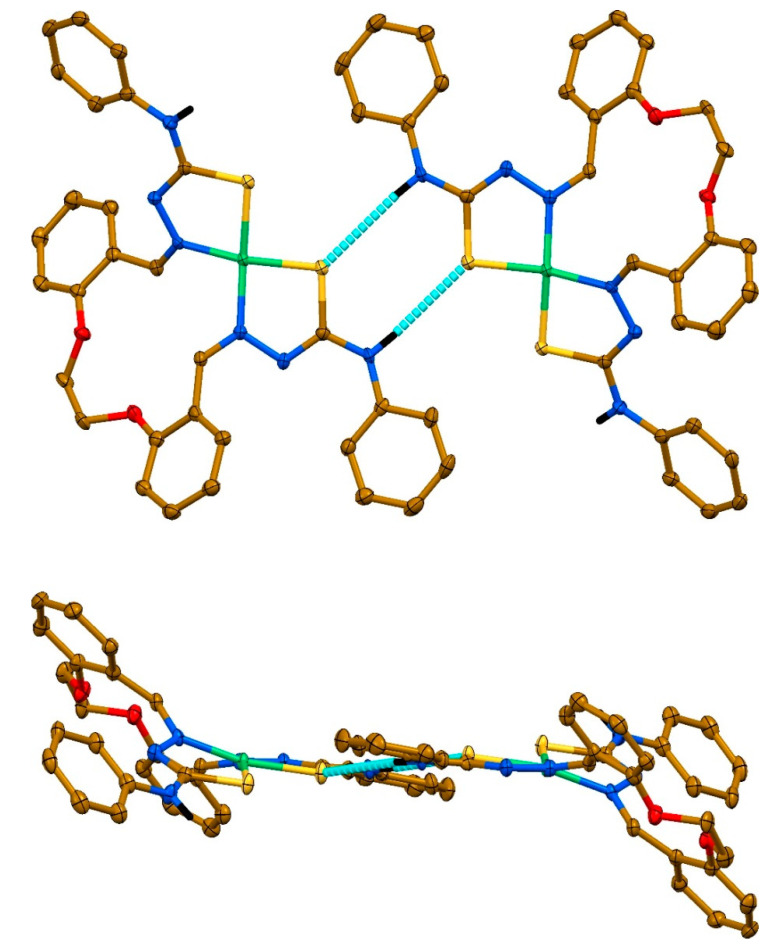
Top and side views on the hydrogen bonded centrosymmetric dimer in the crystal structure of **1**, formed by a pair of molecules **1-I** (ellipsoids are drawn with 40% probability; CH hydrogen atoms are omitted for clarity). A similar centrosymmetric dimer is also formed by a pair of molecules **1-II**. Color code: C = gold, H = black, N = blue, O = red, S = yellow, Ni = green; N–H∙∙∙S hydrogen bond = cyan dashed line.

Complex **1** crystallizes in the triclinic space group *P*–1 with three independent complex molecules, namely **1-I**, **1-II**, and **1-III**, in the asymmetric unit. The metal centers in **1** are bis-chelated by one doubly deprotonated tetradentate ligand **L^I^** in a cis-configuration through two azomethine nitrogen atoms and two thiocarbonyl sulfur atoms, yielding two five-membered metallocycles (Figure 1). Two least-square planes through these metallocycles form a dihedral angle of 20.51(14), 20.42(15), and 22.02(15)° in **1-I**, **1-II**, and **1-III**, respectively, likely dictated for accomplishing coordination of the metal (Table 1). Thus, the nickel(II) cations in the structure of **1** are in a N_2_S_2_ tetracoordinate environment with the formation of a seesaw coordination geometry, as evidenced from the calculated *τ*_4_-descriptors of 0.2146, 0.2206, and 0.2406 in **1-I**, **1-II**, and **1-III**, respectively (Table 1) [15].

The Ni–N and Ni–S bond lengths are pairwise very similar and of 1.914(3)–1.935(3) Å and 2.1389(14)–2.1631(12) Å, respectively (Table 1). The endocyclic chelating N–Ni–S_endocyclic_ bond angles range from 85.59(11)° to 87.04(11)°, while the exocyclic N–Ni–S_exocyclic_ bond angles vary from 162.85(11)° to 168.87(12)° with the most pronounced differences observed in the structure of **1-I** (Table 1). The S–Ni–S bond angle in all the independent molecules of **1** are close to 90° (Table 1). Additionally, the bis(phenoxy)ethane moiety assumes a conformation to avoid steric clashes (Figure 1).

**Figure 2:**
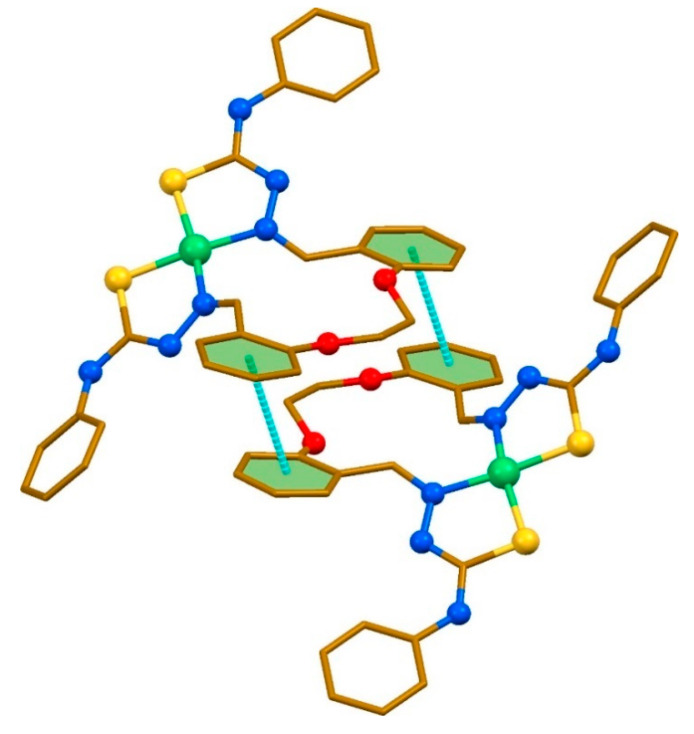
A dimer, formed by π∙∙∙π stacking interactions between two molecules **1-I** in the crystal structure of **1** (hydrogen atoms are omitted for clarity). Color code: C = gold, H = black, N = blue, O = red, S = yellow, Ni = green; π∙∙∙π stacking interaction = cyan dashed line.

Notably, a structurally characterized nickel(II) complex **[Ni(H_2_L^III^)](ClO_4_)_2_∙2MeOH** (**3**) [16] with a similar ligand **H_2_L^III^** [17], but containing the NH_2_ group instead of the PhNH group, was reported, where the phenoxy oxygen donors also participate in coordination towards the metal that adopts a distorted octahedral geometry. However, in the structure of **3**, the parent ligand is coordinated in its neutral form, and Ni(ClO_4_)_2_ was used as a metal source, though trimethylamine was also added in the reaction medium to neutralize the parent organic ligand. Furthermore, in the crystal structure of **1**, two similar centrosymmetric dimers can be revealed (Figure 1), formed by two **1-I** and two **1-II** molecules through a pair of intermolecular N–H∙∙∙S hydrogen bonds (Table 2). The crystal structure of **1** is additionally stabilized by intermolecular π∙∙∙π stacking interactions, formed between the phenylene rings of two molecules **1-I**, also yielding a centrosymmetric dimer (Figure 2, Table 3).

**Figure 3:**
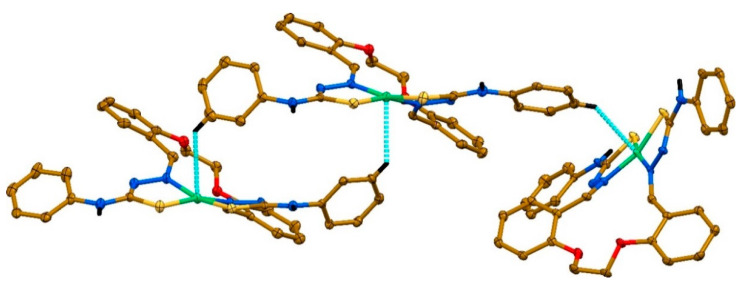
A trimer, formed by the C–H∙∙∙Ni anagostic bonds between molecules **1-III** (**left**), **1-II** (**middle**), and **1-I** (**right**) in the crystal structure of **1** (ellipsoids are drawn with 40% probability; CH hydrogen atoms, not involved in the anagostic interaction, are omitted for clarity). Color code: C = gold, H = black, N = blue, O = red, S = yellow, Ni = green; C–H∙∙∙Ni anagostic interaction = cyan dashed line.

The most striking finding in the crystal structure of **1** is the formation of the so-called anagostic interactions C–H∙∙∙Ni considered in the literature as repulsive forces. Particularly, the nickel(II) cation of **1-I** forms one anagostic bond with one of the phenyl para-hydrogen atoms from an adjacent molecule **1-III**, in which the nickel(II) cation and one of the meta-hydrogen atoms from the other phenyl fragment are also involved in the C–H∙∙∙Ni anagostic interactions with one of the phenyl meta-hydrogen atoms and the metal center of **1-II**, respectively (Figure 3, Table 4). As a result of the mentioned information above, elusive anagostic interactions an asymmetric trimer is formed (Figure 3).

**Figure 4:**
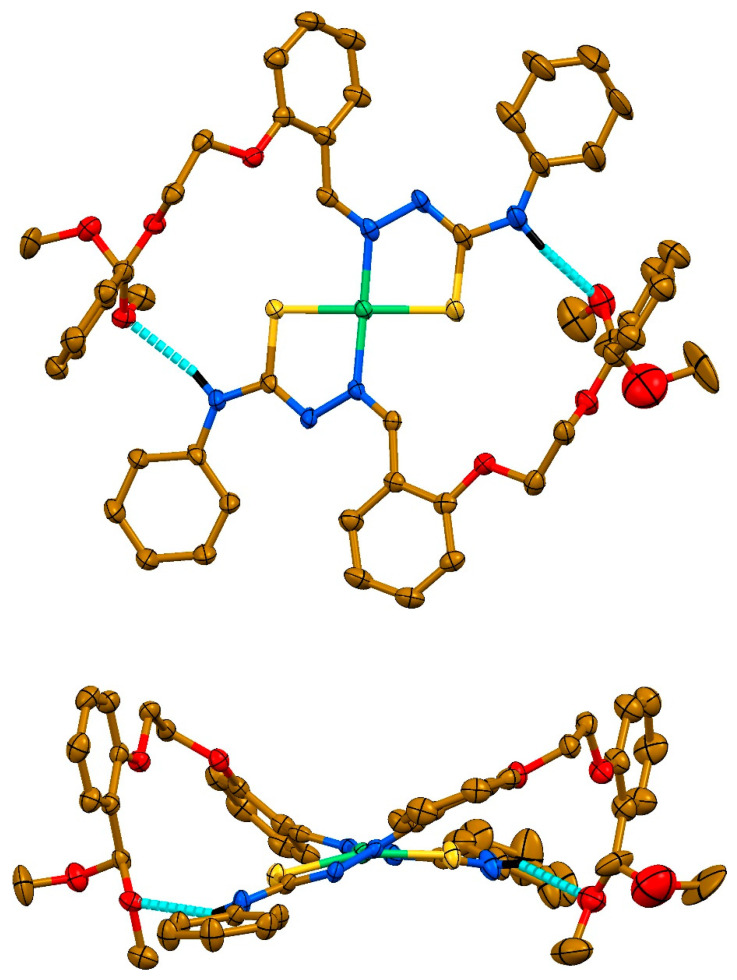
Top and side views on the crystal structure of **2** (ellipsoids are drawn with 40% probability; CH hydrogen atoms are omitted for clarity). Color code: C = gold, H = black, N = blue, O = red, S = yellow, Ni = green; N–H∙∙∙O hydrogen bond = cyan dashed line.

Complex **2** crystallizes in the monoclinic space group *P*2_1_/*c* with one independent complex molecule in the asymmetric unit. The complex shows a pseudo two-fold axis passing through the metal center and normal to the coordination plane (Figure 4). The two organic ligands **L^II^**, which were formed in situ under experimental conditions in their deprotonated form, are coordinated to the metal center through the azomethine nitrogen donors and thiocarbonyl sulfur atoms in a trans-planar configuration, as observed in the majority of the related bis-chelated thiosemicarbazide complexes, also yielding two five-membered metallocycles (Figure 4). Despite two ligands **L^II^** displaying a trans-arrangement in the crystal structure of **2**, the coordination of polyhedron and coordination distances are well-comparable within their esd’s to those measured in the cis-configured disposition of **L^I^** in the crystal structure of **1**. Particularly, in complex **2**, two least-square planes through the five-membered metallocycles also form a very similar dihedral angle of 22.3(2)° (Table 1). The N_2_S_2_ tetracoordinate environment around the metal center also forms a seesaw coordination geometry, as evidenced from the calculated τ_4_-descriptor of 0.1608 (Table 1) [15], which, however, testifies to be closer to a square-planar structure. The Ni–N and Ni–S bond distances as well as the N–Ni–S_endocyclic_ bond angles in the structure of **2** are very similar to those in the molecules of **1** and of about 1.91 and 2.16 Å, and 86°, respectively (Table 1). In the crystal structure of **2**, the N–Ni–N and S–Ni–S bond angles are about 73° larger, while the N–Ni–S_exocyclic_ bond angle is about 70° smaller than those in the crystal structure of **1** (Table 1), which is, obviously, explained by trans- and cis-arrangement of the corresponding donor atoms around the metal center (Figure 1 and Figure 4).

**Figure 5:**
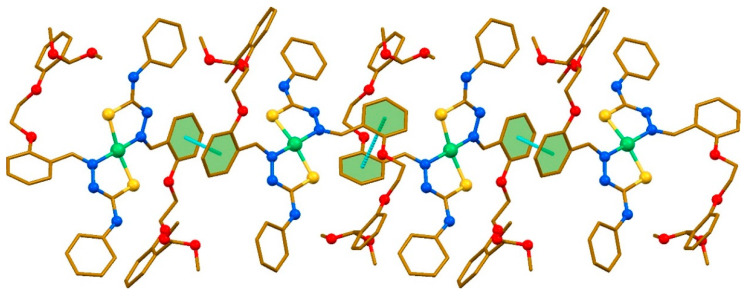
1D polymeric chain, formed along the *c* axis by π⋯π stacking interactions in the crystal structure of **2** (hydrogen atoms are omitted for clarity). Color code: C = gold, H = black, N = blue, O = red, S = yellow, Ni = green; π⋯π stacking interaction = cyan dashed line.

**Figure 6:**
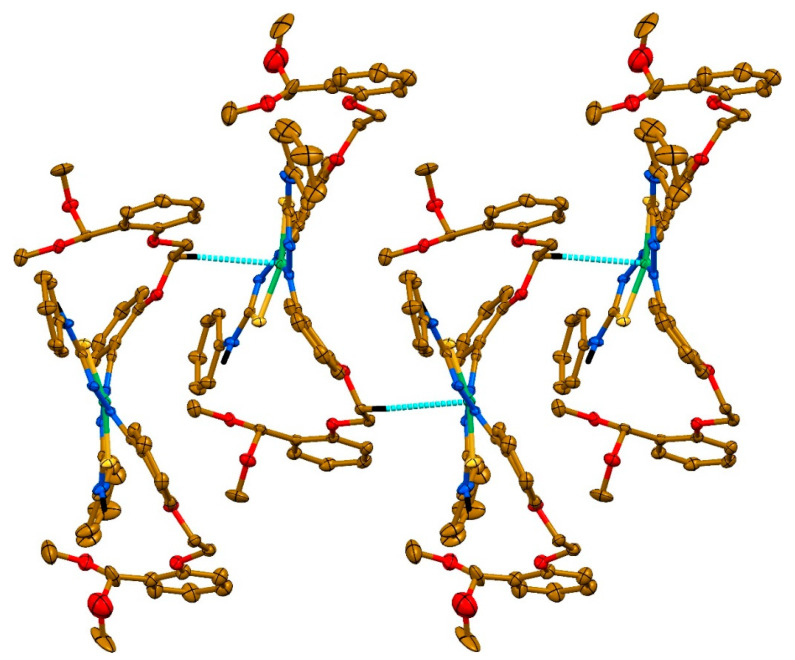
1D polymeric chain, formed along the *b* axis by the C–H∙∙∙Ni anagostic bonds in the crystal structure of **2** (ellipsoids are drawn with 40% probability; CH hydrogen atoms, not involved in the anagostic interaction, are omitted for clarity). Color code: C = gold, H = black, N = blue, O = red, S = yellow, Ni = green; C–H∙∙∙Ni anagostic interaction = cyan dashed line.

The structure of **2** is stabilized by a pair of intramolecular N–H∙∙∙O hydrogen bonds, realized between the NH hydrogen atoms and MeO oxygen atoms (Figure 4, Table 2), thus inducing the multidentate ligands to act as didentate chelating. Furthermore, molecules of **2** are interlinked in a 1D polymeric supramolecular chain along the *c* axis through π∙∙∙π stacking interactions formed by the phenylene rings attached to the imine functions (Figure 5, Table 3). It should be noted that molecules of **2** are further interlinked into a 1D polymeric chain along the *b* axis through the C–H∙∙∙Ni anagostic bonds, formed between the metal centers and one of the CH_2_ hydrogen atoms (Figure 6, Table 4).

We have further applied the Hirshfeld surface analysis [18] to study in detail interactions in the crystal structures of **1** and **2**. As such, associated 2D fingerprint plots [19] were generated using the CrystalExplorer 17.5 software [20]. Notably, since the crystal structure of **1** contains three independent molecules **1-I**, **1-II**, and **1-III**, the data were obtained separately for each of them.

As evidenced from the Hirshfeld surface analysis, the intermolecular H∙∙∙H and H∙∙∙C contacts are major contributors to the crystal packing of all the discussed molecules despite a variety of donor heteroatoms (Table 5). Interestingly, while a proportion of the H∙∙∙H contacts in the molecular surface of **1-II** and **1-III** is almost the same and of about 40%, the same contacts occupy about 47% of the surface in **1-I** and an even higher proportion of about 55% in the surface of **2** (Table 5). The latter can obviously be explained by the presence of two ligands, thus containing a double set of aliphatic and aromatic hydrogen atoms, as well as by the incorporation of the MeO groups in the structure of **2** (Figure 4). The shortest H∙∙∙H contacts are shown in the corresponding fingerprint plots of all molecules as characteristic broad spikes at *d*_e_ + *d*_i_ ≈ 2.0–2.2 Å (Figures S1–S4 in the Supporting Information). It should be noted that a subtle feature is evident in the fingerprint plot of **2**. Particularly, a clear splitting of the short H∙∙∙H fingerprint is observed (Figure S4 in the Supporting Information), which occurs when the shortest contact is between three atoms, rather than for a direct two atom contact [18]. It was also found that intermolecular H∙∙∙C contacts occupy almost the same proportion of about 25% of the Hirshfeld molecular surface of molecules **1-I**, **1-II**, and **2**, while a remarkably higher proportion of the same contacts of about 30% was found in the molecular surface of **1-III** (Table 5). The shortest H∙∙∙C contacts are shown in the corresponding fingerprint plots of all molecules at *d*_e_ + *d*_i_ ≈ 2.5–2.7 Å (Figures S1–S4 in the Supporting Information).

It should also be added that the corresponding 2D fingerprint plots of all the reported molecules contain a significant number of points at large *d*_e_ and *d*_i_, shown as tails at the top right of the plot (Table 5). This is similar to that observed in the fingerprint plots of benzene [18] and phenyl-containing compounds, [21,22,23,24,25,26] and correspond to regions on the Hirshfeld molecular surface without any close contacts to nuclei in adjacent molecules.

The structures of all molecules are also dictated by the intermolecular H∙∙∙N contacts, comprising from 5.5% to 8.1%, as well as by the H∙∙∙S contacts in **1-I**, **1-II**, and **1-III**, and H∙∙∙O contacts in **2** (Table 5). Notably, the H∙∙∙S contacts in **1-I** and **1-III** occupy about 10% of the molecular surface, while a remarkably higher proportion (14%) of the same contacts was found on the Hirshfeld surface of **1-II**. Contrarily, only a minor proportion of the H∙∙∙O and H∙∙∙S contacts was found in the structures of molecules of **1** and **2**, respectively, comprising 1.4–3.0% (Table 5).

Furthermore, all the molecules are also characterized by a significant proportion of the C∙∙∙C contacts, comprising 3.3–5.0% (Table 5). These contacts are shown as the area at *d*_e_ = *d*_i_ ≈ 1.7–2.2 Å in the corresponding 2D fingerprint plots and correspond to π∙∙∙π interactions (Figures S1–S4 in the Supporting Information).

Additionally, it is worth mentioning that the contribution to the total Hirshfeld surface area of all molecules arises from the Ni∙∙∙H contacts being 1.4%, 2.3%, and 0.8% for **1-I**, **1-II** and **1-III**, and **2**, respectively. Notably, these contacts are exclusively shown in the corresponding 2D fingerprint plot of **1-I** as Ni∙∙∙H contacts but no as reciprocal contacts (Figure S1 in the Supporting Information). This is explained by the fact that in the structure of **1-I**, only the metal center is involved in the formation of the intermolecular anagostic bond, while molecules **1-II** and **1-III** each form this type of interactions by both their metal center and one of the hydrogen atoms (Figure 3). The shortest Ni∙∙∙H contacts are shown at *d*_e_ + *d*_i_ ≈ 2.7–2.9 Å in the 2D fingerprint plots of **1-I**, **1-II** and **1-III**, and **2** (Figures S1–S4 in the Supporting Information).

Finally, the structures of all molecules are also described by a negligible proportion of the intermolecular C∙∙∙X and N∙∙∙X contacts, comprising 0.1–2.1% (Table 5, Figures S1–S4 in the Supporting Information).

We additionally calculated the enrichment ratios (*E*) [27] of the intermolecular contacts in order to estimate the propensity of two chemical species to be in contact. All the H∙∙∙X contacts, except the H∙∙∙O and Ni∙∙∙H contacts in **1-III** and **2**, respectively, are favored in the structures of all molecules since the corresponding enrichment ratios *E*_HX_ are close to or even higher than unity (Table 5). The C∙∙∙C contacts in the structures of **1-I**, **1-II**, and **2** are highly enriched (*E*_CC_ = 1.25–1.43), while the same contacts in the structure of **1-III** are significantly less favored (*E*_CC_ = 0.77), although the *S*_C_ value of the structure of **1-III** is the highest among all the discussed molecules. This is related to the high proportion and enrichment of H∙∙∙C contacts (Table 5). Remaining contacts are significantly impoverished (Table 5).

In order to provide deeper insight into the nature of physical factors and non-covalent interactions, which influence the stability of the reported metal complexes, the ETS-NOCV [28] charge and energy decomposition method were applied as implemented in the ADF package [29,30]. We applied DFT/BLYP-D3/TZP since these types of computational details provide reliable results for non-covalent interactions [31,32].

**Figure 7:**
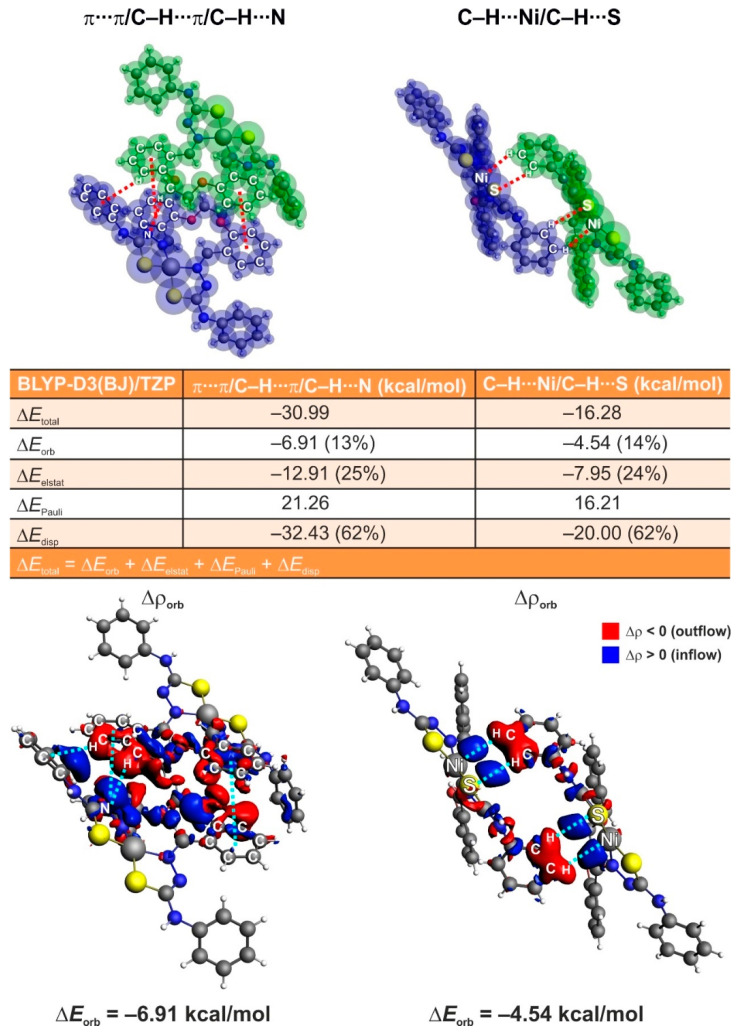
The results of ETS-NOCV energy decomposition describing π∙∙∙π, C–H∙∙∙π and C–H∙∙∙N (**left**), and C–H∙∙∙Ni and C–H∙∙∙S (**right**) interactions in **1**. Additionally, the overall deformation density Δρ_orb_ with the corresponding Δ*E*_orb_ are presented.

**Figure 8:**
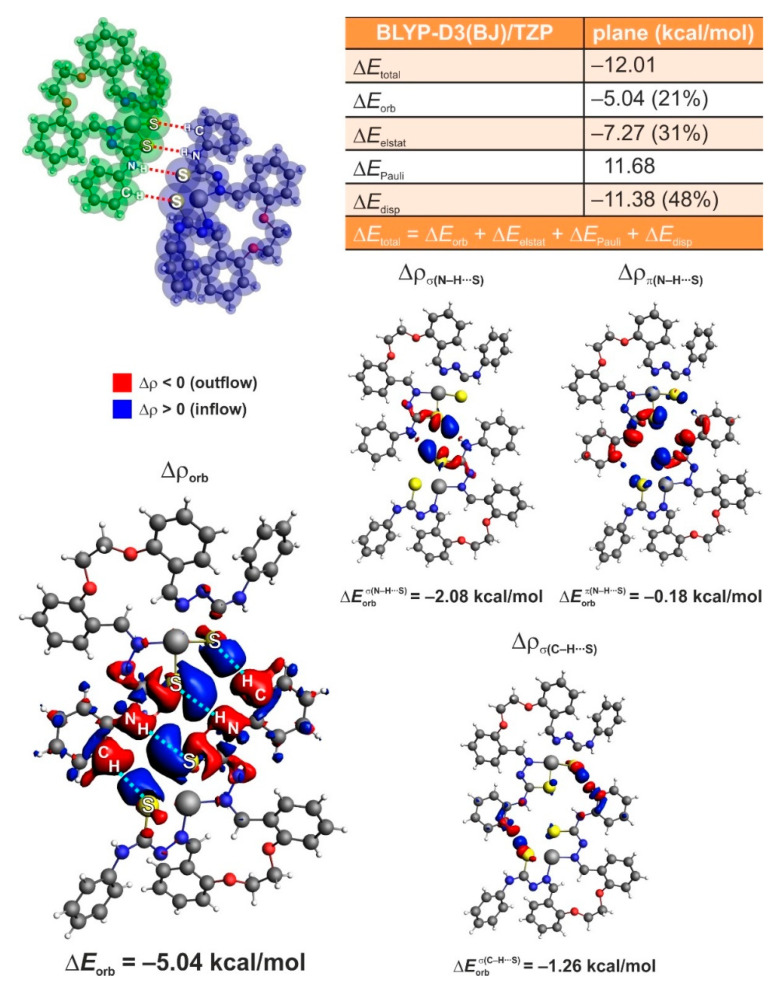
The results of ETS-NOCV energy decomposition describing N–H∙∙∙S and C–H∙∙∙S interactions between in-plane monomers in **1**. Additionally, the overall deformation density Δρ_orb_ with the corresponding Δ*E*_orb_ are presented together with σ- and π-NOCV based contributions.

As it was already mentioned, one of the most intriguing and elusive contributors to self-assembling of **1** and **2** is a long range (2.71–2.94 Å) C–H∙∙∙Ni contact (Figure 3 and Figure 7, Table 4), which is considered in the literature as a repulsive term based rather on chemical intuition without any computational or experimental proofs. The ETS-NOCV results of **1** unveiled that cooperative action of both long-range C–H∙∙∙Ni and C–H∙∙∙S interactions leads to the very low dimerization energy, Δ*E*_total_ = –16.28 kcal/mol, caused chiefly by the London dispersion term (Figure 7). Surprisingly, it is even more efficient than more intuitive in-plane σ-type hydrogen bonds N–H∙∙∙S and C–H∙∙∙S, further supported by π-delocalizations (Figure 8) [33,34,35]. Furthermore, there are clearly charge delocalizations discovered from the contour of Δρ_orb_ stemming from the two ways transfers in C–H∙∙∙Ni: [Ni(d_z_^2^) → σ*(C–H) and σ(C–H) → Ni(d_z_^2^)] and within C–H∙∙∙S [S(Lp) → σ*(C–H)] (Figure 7).

Since both C–H∙∙∙Ni and C–H∙∙∙S interactions are present in **1**, and ETS-NOCV cannot separate their individual strengths as indicated by the contour Δρ_orb_, we decided to perform additionally an Interacting Quantum Atoms (IQA) [36] energy decomposition based study which allows us to discern Ni∙∙∙H vs. S∙∙∙H (Table 6). The obtained results nicely point out the importance of sizeable stabilization stemming from both Ni∙∙∙H (Δ*E*_int_ = –9.71 kcal/mol) and S∙∙∙H (Δ*E*_int_ = –2.87 kcal/mol), despite their long distances of about 2.84 Å, where the repulsion could be expected [37,38] (Table 6). We noticed similar stabilizations for the intramolecular C–H∙∙∙Ni contacts in other complexes based on thiourea derived ligands [37]. Interestingly, the Ni∙∙∙H interactions are dominated by the attractive Coulomb term, Δ*E*_Coulomb_ = –6.37 kcal/mol, followed by the exchange-correlation constituent Δ*E*_XC_ = –3.33 kcal/mol, whereas the S∙∙∙H interactions are characterized by the repulsive Coulomb forces, Δ*E*_Coulomb_ = 1.94 kcal/mol, and the sole attractive force in S∙∙∙H which is Δ*E*_XC_ = – 4.80 kcal/mol (Table 6). It should be added that intermolecular Ni∙∙∙H interactions described herein are stronger than the intramolecular ones reported by us recently [37]. Both intra- and intermolecular Ni∙∙∙H interactions are constituted from prevailing attractive Coulomb forces followed by the exchange-correlation constituent (Table 6) [37].

**Figure 9:**
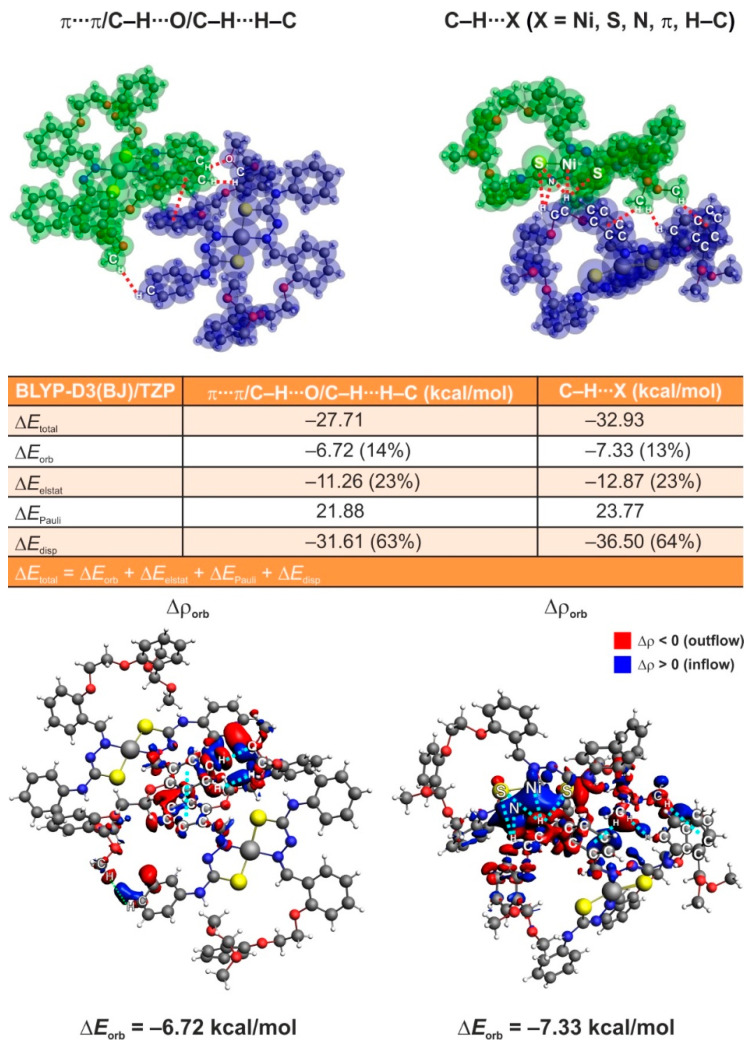
The results of ETS-NOCV energy decomposition describing the cooperativity of π∙∙∙π, C–H∙∙∙O and C–H∙∙∙H–C (**left**), and C–H∙∙∙X (X = Ni, S, N, π, H–C) (**right**) interactions in **2**. Additionally, the involvement of the nickel(II) d-orbitals is depicted.

**Figure 10:**
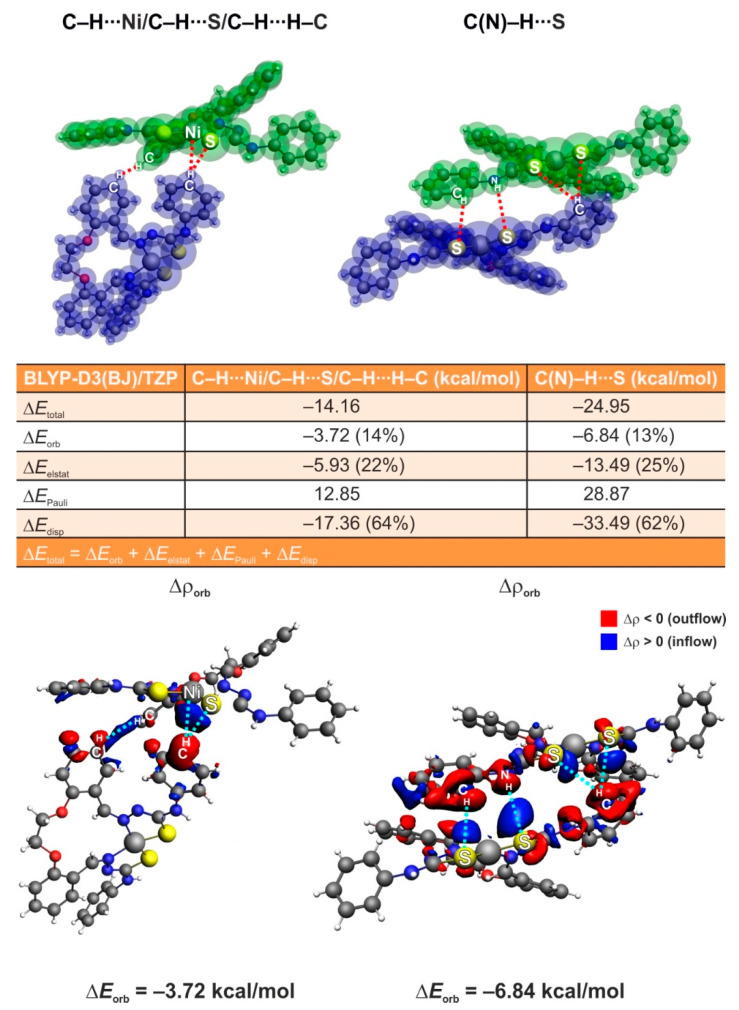
The results of ETS-NOCV energy decomposition describing C–H∙∙∙Ni, C–H∙∙∙S and C–H∙∙∙H–C (**left**), and C–H∙∙∙S and N–H∙∙∙S (**right**) interactions in **1**. Additionally, the overall deformation density Δρ_orb_ with the corresponding Δ*E*_orb_ are presented.

As far as **2** is considered, it is seen that the cooperativity of the C–H∙∙∙Ni and less intuitive C–H∙∙∙X (X = S, N, π, H–C) interactions [37,38,39,40,41,42,43,44,45,46] provides the most efficient stabilization with Δ*E*_total_ = –32.93 kcal/mol (Figure 9), which is stronger with respect to **1** (Figure 7 and Figure 10). Stabilization stemming from π∙∙∙π and C–H∙∙∙X (X = O, H–C) interactions in **2** (Figure 9) is less efficient than in **1** (Figure 7). It is to be noted that the presence of supportive, recently topical homopolar C–H∙∙∙H–C interactions, discussed in terms of in-depth understanding of steric-crowding [39,40,41,42,43,44,45] (Figure 9 and Figure 10), explains also the Hirshfeld based observation on the dominance of H∙∙∙H contacts in the reported crystal structures.

**Figure 11:**
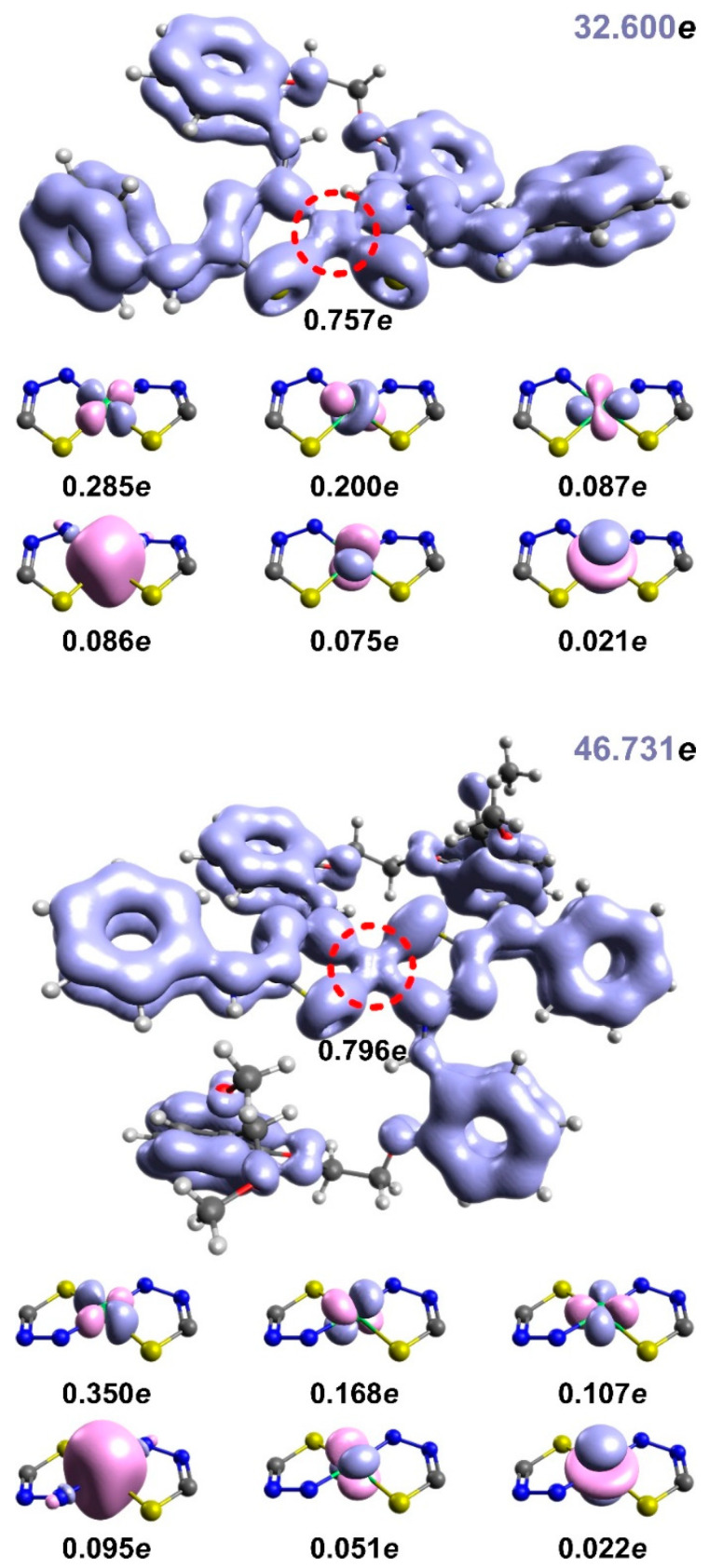
EDDB(r) contours together with populations for **1** (**top**) and **2** (**bottom**). Additionally, the involvement of the nickel(II) d-orbitals is depicted.

We finally studied the aromaticity by the electron density of delocalized bonds (EDDB) method [47], which is suitable for both qualitative and quantitative analyses of electrons’ delocalization in various aromatic compounds, including very challenging metal complexes. It is established that in both complexes **1** and **2**, the extended π-delocalizations are observed mostly at the phenyl units and the adjacent HCNN linkers (Figure 11). Interestingly, the σ-delocalization starts to dominate when going to the metal proximity (Figure 11). It proves that nickel(II) is conjugated to the neighboring atoms predominantly through σ-channels. Furthermore, in both cases, it is very weak conjugation since only about 0.8|e| is delocalized through the Ni–N and Ni–S bonds. Further analyses revealed consistently that mostly in-plane d-orbitals are involved in σ-conjugation (Figure 11).

## 3. Materials and Methods

### 3.1. Materials

Unless stated otherwise, all chemicals were obtained from Sigma-Aldrich, and were used as received.

### 3.2. Physical Measurements

Microanalyses were performed using a Heraeus CHN-O-Rapid analyser (Heraeus, Hanau, Germany). The FTIR spectra were recorded on a Bruker Tensor 27 FTIR spectrometer (Bruker, Karlsruhe, Germany).

### 3.3. Synthesis of Complexes

Complexes were synthesized using a branched tube method [48]. A mixture of **H_2_L^I^** (0.284 g, 0.5 mmol) and Ni(CH_3_COO)_2_·2H_2_O (0.106 g, 0.5 mmol) were placed in the main arm of a branched tube. EtOH or MeOH (15 mL) was carefully added to fill the arms. The tube was sealed and immersed in an oil bath at 60 °C while the branched arm was kept at ambient temperature. After few days, X-ray suitable single crystals of the corresponding complex were formed in the cooler arm of the tube. Crystals were isolated by filtration.

**[NiL^I^] (1)**. Block-like crystals. Isolated yield: 0.225 g (72%). Anal. Calc. for C_30_H_26_N_6_NiO_2_S_2_ (625.39): C 57.62, H 4.19 and N 13.44; found: C 57.51, H 4.28 and N 13.53%.

**[Ni(L^II^)_2_] (2)**. Plate-like crystals. Isolated yield: 0.222 g (45%). Anal. Calc. for C_50_H_52_N_6_NiO_8_S_2_ (987.81): C 60.80, H 5.31 and N 8.51; found: C 60.71, H 5.43 and N 8.62%.

### 3.4. Single-Crystal X-ray Diffraction

Diffraction data for **1** were collected on a Bruker Smart Apex II diffractometer equipped with CCD, and those of **2** on a Enraf Nonius CAD4. Both the experiments were performed at 100 K with Mo-Kα radiation (λ = 0.71073 Å). Cell refinement, indexing, and scaling of the data sets were carried out using the Mosflm, Denzo/HKL suite [49,50] and Bruker Smart Apex and Saint packages [51]. The structures were solved by direct methods and subsequent Fourier analyses and refined by the full-matrix least-squares method based on *F*^2^ with all observed reflections [52]. The contribution of hydrogen atoms in complexes was introduced in the final cycles of refinement at the calculated position, except those of the imino nitrogen atoms in **2**, located on the Fourier map. All the calculations were performed using the WinGX System, Ver 2013.13 [53].

Crystal data for **1.** C_30_H_26_N_6_NiO_2_S_2_, M_r_ = 625.40 g mol^−1^, triclinic, space group P–1, a = 16.2571(11), b = 17.0000(12), c = 17.4769(12) Å, α = 86.288(5), β = 64.780(4), γ = 71.536(5)°, V = 4130.9(5) Å^3^, Z = 6, ρ = 1.508 g cm^−3^, μ(Mo-Kα) = 0.897 mm^−1^, reflections: 15090 collected, 15090 unique, R_int_ = 0.048, R_1_(all) = 0.0817, wR_2_(all) = 0.1258, S = 1.066.

Crystal data for **2.** C_50_H_52_N_6_NiO_8_S_2_, M_r_ = 987.80 g mol^−1^, monoclinic, space group P2_1_/c, a = 19.014(2), b = 12.4932(15), c = 21.984(3) Å, β = 115.557(4)°, V = 4711.3(10) Å^3^, Z = 1, ρ = 1.393 g cm^−3^, μ(Mo-Kα) = 0.562 mm^−1^, reflections: 46736 collected, 5757 unique, R_int_ = 0.104, R_1_(all) = 0.1057, wR_2_(all) = 0.1819, S = 1.065.

CCDC 1998447 and 1998448 contain the supplementary crystallographic data. These data can be obtained free of charge via http://www.ccdc.cam.ac.uk/conts/retrieving.html, accessed on 17 March 2021, or from the Cambridge Crystallographic Data Centre, 12 Union Road, Cambridge CB2 1EZ, UK; fax: (+44) 1223-336-033; or e-mail: deposit@ccdc.cam.ac.uk.

### 3.5. ETS-NOCV Studies

In order to shed light on the nature of bonding, the charge and energy decomposition scheme ETS-NOCV [28] was applied as implemented in the ADF package [29,30]. This approach allows us to understand chemical bonding in terms of qualitative and quantitative delineation of various bonding channels (σ, π, etc.), and it also decomposes total interaction energy (Δ*E*_total_) into physically meaningful contributions: Δ*E*_total_ = Δ*E*_orb_ + Δ*E*_elstat_ + Δ*E*_Pauli_ + Δ*E*_disp_. The orbital interaction term Δ*E*_orb_ (corresponding to Δρ_orb_ = $\underset{i}{\sum }\Delta \rho \left(i\right)$ ) covers various charge delocalizations/contributions Δρ_orb_(i) (σ, π, etc.), further supplemented by the corresponding energies Δ*E*_orb_(i) for any system even without symmetry. The second term, Δ*E*_elstat_, represents the classical electrostatic interaction between the selected subsystems. The next term, Δ*E*_Pauli_, concerns Pauli repulsion between occupied orbitals of fragments. Finally, the last contribution, Δ*E*_disp_, corresponds to the semi-empirical Van der Waals component. It shall be added that overall interaction energies are calculated not from the supermolecular approach, but according to the ETS method [28].

### 3.6. IQA studies

The Interacting Quantum Atoms Energy decomposition scheme (IQA) [36] operates in atomic resolution as opposed to the ETS-NOCV. It allows us to approximate overall system energy by a sum of atomic and diatomic contributions, where the latter can be in turn decomposed into physically relevant Coulomb and exchange-correlation constituents: Δ*E*_int_ = Δ*E*_Coulomb_ + Δ*E*_XC_.

### 3.7. EDDB studies

The EDDB(r) quantity is a part of electron density (ED) ED(r) = EDLA(r) + EDLB(r) + EDDB(r), where EDLA represents electrons localized on atoms (inner shells, lone pairs); EDLB represents electrons in Lewis-like localized bonds; and EDDB represents electrons delocalized between conjugated bonds (multicenter electron sharing, aromatic rings) [47]. The latter is calculated based on diatomic blocks of a charge and bond-order matrix.

## 4. Conclusions

In summary, two novel discrete mononuclear homoleptic complexes of the nickel(II) cation were synthetized using a one-pot synthetic approach, and extensively characterized by both experimental and theoretical approaches. Complex **[NiL^I^]** (**1**) was obtained in ethanol from the bis-thiosemicarbazone ligand (**H_2_L^I^**), prepared by the condensation of 4-phenylthiosemicarbazide with bis-aldehyde, namely 2,2′-(ethane-1,2-diylbis(oxy)dibenzaldehyde, and contains three independent molecules, namely **1-I**, **1-II**, and **1-III**, in the asymmetric unit. Complex **[Ni(L^II^)_2_]** (**2**), where **L^II^** is a monodeprotonated ligand 2-(2-(2-(2-(dimethoxymethyl)phenoxy)ethoxy)benzylidene)-N-phenylhydrazine-1-carbothioamide (**HL^II^**), was formed using the same synthetic approach and precursors but in methanol. Thus, the ligand **L^II^** was formed in situ from the reaction of **L^I^** with methanol upon coordination to the metal center under synthetic conditions. The doubly deprotonated ligand **L^I^** in **1** is coordinated in a cis-manner, while two ligands **L^II^** are coordinated in a trans-manner in **2**, both yielding an N_2_S_2_ coordination environment, formed by the azomethine nitrogen atoms and the thiocarbonyl sulfur atoms, with a seesaw coordination polyhedron around the metal centers.

It was determined based on the charge and energy decomposition scheme ETS-NOCV that supramolecular networks in the reported structures are due to cooperative action of mostly London dispersion dominated N–H∙∙∙S and N–H∙∙∙O hydrogen bonds in **1** and **2**, respectively, and a bunch of efficient C–H∙∙∙X (where X = S, N, O, π, H–C), π∙∙∙π stacking and the most elusive long-range (~2.8 Å), attractive C–H∙∙∙Ni preagostic as well as recently topical homopolar dihydrogen C–H∙∙∙H–C [39,40,41,42] interactions. It was further unveiled that the intermolecular preagostic C–H∙∙∙Ni interactions are constituted from both stabilizing Coulomb forces and an exchange-correlation contribution (contrary to the literature claims on its pure Coulombic and repulsive character [54]) as opposed to the analogous long-range C–H∙∙∙S interactions, where the attraction stems from the dominant exchange-correlation contribution over the repulsive Coulomb component. The electron density of delocalized bonds (EDDB) method demonstrates that the nickel(II) cation is involved in weak σ-conjugation with the adjacent in-plane atoms since only ~0.8|e| are delocalized through the system of Ni–N and Ni–S bonds, which suggests a very weak σ-metalloaromatic character.

Finally, complex **2** might be of particular interest as a complex agent, containing two podand-like functions, which potentially can trap suitable species; thus, it can be used, e.g., in membrane transport and liquid-liquid extraction. These comprehensive studies are currently in progress and will be reported elsewhere in the case of successful results.

## Figures and Tables

**Table 1:** Selected Bond Lengths (Å) and Angles (°) for **1** and **2**.

	1-I	1-II	1-III	2
Bond lengths				
Ni–N	1.914(3) 1.928(4)	1.918(3) 1.923(3)	1.916(3) 1.935(3)	1.903(4) 1.907(4)
Ni–S	2.1464(14) 2.1506(11)	2.1502(13) 2.1631(12)	2.1389(14) 2.1453(14)	2.160(2) 2.161(2)
Bond angles				
N–Ni–N	101.26(15)	100.27(14)	102.20(14)	173.1(2)
N–Ni–S_endocyclic_	85.59(11) 86.62(10)	86.14(11) 86.46(11)	86.53(11) 87.04(11)	85.94(19) 86.12(19)
N–Ni–S_exocyclic_	163.87(11) 168.87(12)	163.98(11) 164.92(11)	162.85(11) 163.23(11)	94.90(19) 94.95(19)
S–Ni–S	89.90(5)	90.96(5)	88.39(5)	164.23(8)
Dihedral angle				
NiNNCS∙∙∙ NiNNCS	20.51(14)	20.42(15)	22.02(15)	22.3(2)

**Table 2:** Hydrogen Bond Lengths (Å) and Angles (°) for **1** and **2**.

	D–H∙∙∙A	*d*(D–H)	*d*(H∙∙∙A)	*d*(D∙∙∙A)	∠(DHA)
**1**	N1–H1N∙∙∙S1	0.88	2.80	3.672(4)	175
	N12–H12N∙∙∙S4	0.88	2.79	3.482(3)	136
**2**	N1–H1N∙∙∙O7	0.86(7)	2.12(7)	2.922(9)	154(7)
	N4–H4N∙∙∙O4	0.89(7)	2.14(8)	2.949(7)	152(6)

**Table 3:** π⋯π Distances (Å) and Angles (°) for **1** and **2** ^1^.

	Cg(*I*)	Cg(*J*)	*d*[Cg(*I*)–Cg(*J*)]	*α*	*β*	*γ*	Slippage
**1**	C_6_H_4_	C_6_H_4_	3.728(3)	1.3(2)	24.3	25.3	1.534
	C_6_H_4_	C_6_H_4_	3.729(3)	1.3(2)	25.3	24.3	1.595
**2**	C_6_H_4_	C_6_H_4_	3.853(4)	12.4(4)	13.8	16.3	0.918
	C_6_H_4_	C_6_H_4_	3.854(4)	12.4(4)	16.3	13.8	1.083

^1^ Cg(*I*)–Cg(*J*): distance between ring centroids; *α*: dihedral angle between planes Cg(*I*) and Cg(*J*); *β*: angle Cg(*I*) → Cg(*J*) vector and normal to plane I; *γ*: angle Cg(*I*) → Cg(*J*) vector and normal to plane *J*; slippage: distance between Cg(*I*) and perpendicular projection of Cg(*J*) on ring *I*.

**Table 4:** Bond Lengths (Å) and Angles (°) for **1** and **2**.

	1-I	1-II	1-III	2
Bond lengths				
C–H	0.95	0.95	0.95	0.99
Ni∙∙∙H	2.71	2.94	2.84	2.91
Ni∙∙∙C	3.504(5)	3.657	3.563	3.713
Bond angle				
Ni∙∙∙H–C	142	133	133	139

**Table 5:** Hirshfeld Contact Surfaces and Derived “Random Contacts” and “Enrichment Ratios” for **1-I**, **1-II**, **1-III**, and **2**.

	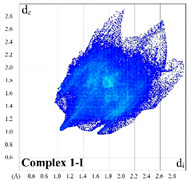						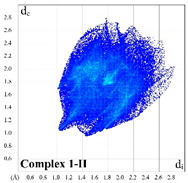						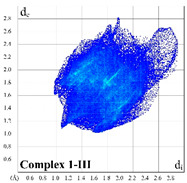						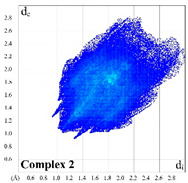					
	H	C	N	O	S	Ni	H	C	N	O	S	Ni	H	C	N	O	S	Ni	H	C	N	O	S	Ni
Contacts (*C*, %) ^1^																								
H	47.3	–	–	–	–	–	38.9	–	–	–	–	–	39.6	–	–	–	–	–	54.7	–	–	–	–	–
C	26.2	5.0	–	–	–	–	24.4	4.6	–	–	–	–	30.4	3.3	–	–	–	–	24.0	3.5	–	–	–	–
N	6.9	0.4	0.0	–	–	–	8.1	1.5	0.1	–	–	–	7.5	1.4	0.0	–	–	–	5.5	0.8	0.0	–	–	–
O	2.3	0.7	0.0	0.0	–	–	3.0	1.7	0.0	0.0	–	–	1.4	2.1	0.0	0.0	–	–	6.6	0.4	0.2	0.0	–	–
S	9.8	0.1	0.0	0.0	0.0	–	14.0	0.1	0.2	0.0	0.3	–	9.9	0.1	0.2	0.0	0.9	–	2.5	0.1	0.0	0.2	0.0	–
Ni	1.4	0.0	0.0	0.0	0.0	0.0	2.3	0.7	0.1	0.0	0.0	0.0	2.3	0.8	0.1	0.0	0.0	0.0	0.8	0.8	0.0	0.0	0.0	0.0
Surface (*S*, %)																								
	70.6	18.7	3.7	1.5	5.0	0.7	64.8	18.8	5.1	2.4	7.5	1.6	65.4	20.7	4.6	1.8	6.0	1.6	74.4	16.6	3.3	3.7	1.4	0.8
Random contacts (*R*, %)																								
H	49.8	–	–	–	–	–	42.0	–	–	–	–	–	42.8	–	–	–	–	–	55.4	–	–	–	–	–
C	26.4	3.5	–	–	–	–	24.4	3.5	–	–	–	–	27.1	4.3	–	–	–	–	24.7	2.8	–	–	–	–
N	5.2	1.4	0.1	–	–	–	6.6	1.9	0.3	–	–	–	6.0	1.9	0.2	–	–	–	4.9	1.1	0.1	–	–	–
O	2.1	0.6	0.1	0.0	–	–	3.1	0.9	0.2	0.1	–	–	2.4	0.7	0.2	0.0	–	–	5.5	1.2	0.2	0.1	–	–
S	7.1	1.9	0.4	0.2	0.3	–	9.7	2.8	0.8	0.4	0.6	–	7.8	2.5	0.6	0.2	0.4	–	2.1	0.5	0.1	0.1	0.0	–
Ni	1.0	0.3	0.1	0.0	0.1	0.0	2.1	0.6	0.2	0.1	0.2	0.0	2.1	0.7	0.1	0.1	0.2	0.0	1.2	0.3	0.1	0.1	0.0	0.0
Enrichment (*E*) ^2^																								
H	0.95	–	–	–	–	–	0.93	–	–	–	–	–	0.93	–	–	–	–	–	0.99	–	–	–	–	–
C	0.99	1.43	–	–	–	–	1.00	1.31	–	–	–	–	1.12	0.77	–	–	–	–	0.97	1.25	–	–	–	–
N	1.33	0.29	–	–	–	–	1.23	0.79	–	–	–	–	1.25	0.74	–	–	–	–	1.12	0.73	–	–	–	–
O	1.10	–	–	–	–	–	0.97	–	–	–	–	–	0.58	–	–	–	–	–	1.20	0.33	–	–	–	–
S	1.38	0.05	–	–	–	–	1.44	0.04	–	–	–	–	1.27	0.04	–	–	–	–	1.19	–	–	–	–	–
Ni	1.40	–	–	–	–	–	1.10	–	–	–	–	–	1.10	–	–	–	–	–	0.67	–	–	–	–	–

^1^ Values are obtained from CrystalExplorer 17.5 [20]. ^2^ The “enrichment ratios” were not computed when the “random contacts” were lower than 0.9%, as they are not meaningful [27].

**Table 6:** IQA Energy Decomposition of the Diatomic Long-range Ni∙∙∙H and S∙∙∙H Interactions in **1** (the model from Figure 7 is considered).

IQA/BLYP/6-311+G(d,p)	Δ*E*_int_^1^	Δ*E*_Coulomb_	Δ*E*_XC_
Ni∙∙∙H (2.843 Å)	–9.71	–6.37	–3.33
S∙∙∙H (2.840 Å)	–2.87	1.94	–4.80

^1^ Δ*E*_int_ = Δ*E*_Coulomb_ + Δ*E*_XC_ [36].

## Data Availability

All the data supporting the conclusions is included within the manuscript and is available on request from the corresponding authors.

## Supplementary Materials

The following are available online at https://www.mdpi.com/1422-0067/22/10/5337/s1. 2D and decomposed 2D fingerprint plots of observed contacts for **1-I**, **1-II**. **1-III** and **2**.
