# Correction: Mahmoudi et al. Solvent-Induced Formation of Novel Ni(II) Complexes Derived from Bis-Thiosemicarbazone Ligand: An Insight from Experimental and Theoretical Investigations. *Int. J. Mol. Sci.* 2021, *22*, 5337

**DOI:** 10.3390/ijms232113410

**Published:** 2022-11-02

**Authors:** Ghodrat Mahmoudi, Maria G. Babashkina, Waldemar Maniukiewicz, Farhad Akbari Afkhami, Bharath Babu Nunna, Fedor I. Zubkov, Aleksandra L. Ptaszek, Dariusz W. Szczepanik, Mariusz P. Mitoraj, Damir A. Safin

**Affiliations:** 1 Department of Chemistry, Faculty of Science, University of Maragheh, Maragheh P.O. Box 55181-83111, Iran; 2 Independent Researcher, Respubliki Str. 14, 625003 Tyumen, Russia; 3 Institute of General and Ecological Chemistry, Lodz University of Technology, Zeromskiego 116, 90-924 Łódź, Poland; 4 Department of Chemistry, The University of Alabama, Box 870336, 250 Hackberry Lane, Tuscaloosa, AL 35487, USA; 5 Department of Mechanical and Industrial Engineering, New Jersey Institute of Technology, University Heights, Newark, NJ 07102, USA; 6 Department of Medicine, Division of Engineering in Medicine, Brigham and Women’s Hospital, Harvard Medical School, Harvard University, Cambridge, MA 02139, USA; 7 Organic Chemistry Department, Faculty of Science, Peoples’ Friendship University of Russia (RUDN University), Miklukho-Maklaya Str. 6, 117198 Moscow, Russia; 8 Department of Theoretical Chemistry, Faculty of Chemistry, Jagiellonian University, Gronostajowa 2, 30-387 Krakow, Poland; 9 Institute of Chemistry, University of Tyumen, Volodarskogo Str. 6, 625003 Tyumen, Russia; 10 Innovation Center for Chemical and Pharmaceutical Technologies, Ural Federal University Named after the First President of Russia B.N. Eltsin, Mira Str. 19, 620002 Ekaterinburg, Russia; 11 «Advanced Materials for Industry and Biomedicine» Laboratory, Kurgan State University, Sovetskaya Str. 63/4, 640020 Tyumen, Russia

## Affiliation Correction

We received a complaint from the Université Catholique de Louvain. In the published version of the article [1], there was an error regarding the affiliation for Maria G. Babashkina. The correct affiliation is as follows: Independent Researcher, Respubliki Str. 14, 625003 Tyumen, Russia. The authors apologize for any inconvenience caused, and state that the scientific conclusions are unaffected. The authors agreed with this correction. This correction was approved by the Academic Editor. The original publication has also been updated.
