# Supplementary material for: Solvent-Induced Formation of Novel Ni(II) Complexes Derived from Bis-Thiosemicarbazone Ligand: An Insight from Experimental and Theoretical Investigations
Source: Int J Mol Sci. 2021 May 19;22(10):5337. doi: 10.3390/ijms22105337 (PMC8159110; doi:10.3390/ijms22105337)
Supplement: Supplementary file 1 [file ijms-22-05337-s001.zip › ijms-1193253-supplementary.pdf]

Supporting Information

Solvent-induced Formation of Novel Ni(II) Complexes Derived from Bis-Thiosemicarbazone Ligand: An Insight from Experimental and Theoretical Investigations

Ghodrat Mahmoudi [1,*], Maria G. Babashkina [2], Waldemar Maniukiewicz [3,*], Farhad Akbari Afkhami [4], Bharath Babu Nunna [5], Fedor I. Zubkov [6], Aleksandra L. Ptaszek [7], Dariusz W. Szczepanik [7], Mariusz P. Mitoraj [7,*] and Damir A. Safin [8,9,10,*]

[1] Department of Chemistry, Faculty of Science, University of Maragheh, P.O. Box 55181-83111, Maragheh, Iran; ghodratmahmoudi@gmail.com
[2] Institute of Condensed Matter and Nanosciences, Université Catholique de Louvain, Place L. Pasteur 1, 1348 Louvain-la-Neuve, Belgium; maria.babashkina@gmail.com
[3] Institute of General and Ecological Chemistry, Lodz University of Technology, Żeromskiego 116, 90-924 Łódź, Poland; waldemar.maniukiewicz@p.lodz.pl
[4] Department of Chemistry, The University of Alabama, Box 870336, 250 Hackberry Lane, Tuscaloosa, Alabama 35487, USA; f.a.afkhami@gmail.com
[5] Department of Mechanical and Industrial Engineering, New Jersey Institute of Technology, University Heights, Newark, NJ 07102, USA; bn63@njit.edu
[6] Organic Chemistry Department, Faculty of Science, Peoples' Friendship University of Russia (RUDN University), Miklukho-Maklaya Str. 6, Moscow, 117198, Russian Federation; fzubkov@sci.pfu.edu.ru
[7] Department of Theoretical Chemistry, Faculty of Chemistry, Jagiellonian University, Gronostajowa 2, 30-387 Cracow, Poland; aleksandra.ptaszek6@gmail.com (A.L.P.); dszczpnk@gmail.com (D.W.S.),
[8] University of Tyumen, Volodarskogo Str. 6, 625003 Tyumen, Russian Federation
[9] Innovation Center for Chemical and Pharmaceutical Technologies, Ural Federal University named after the First President of Russia B.N. Eltsin, Mira Str. 19, Ekaterinburg, 620002, Russian Federation
[10] Kurgan State University, Sovetskaya Str. 63/4, 640020, Russian Federation
* Correspondence: ghodratmahmoudi@gmail.com (G.M.); waldemar.maniukiewicz@p.lodz.pl (W.M.); mitoraj@chemia.uj.edu.pl (M.P.M.), mitoraj@chemia.uj.edu.pl (M.P.M.); damir.a.safin@gmail.com (D.A.S.)

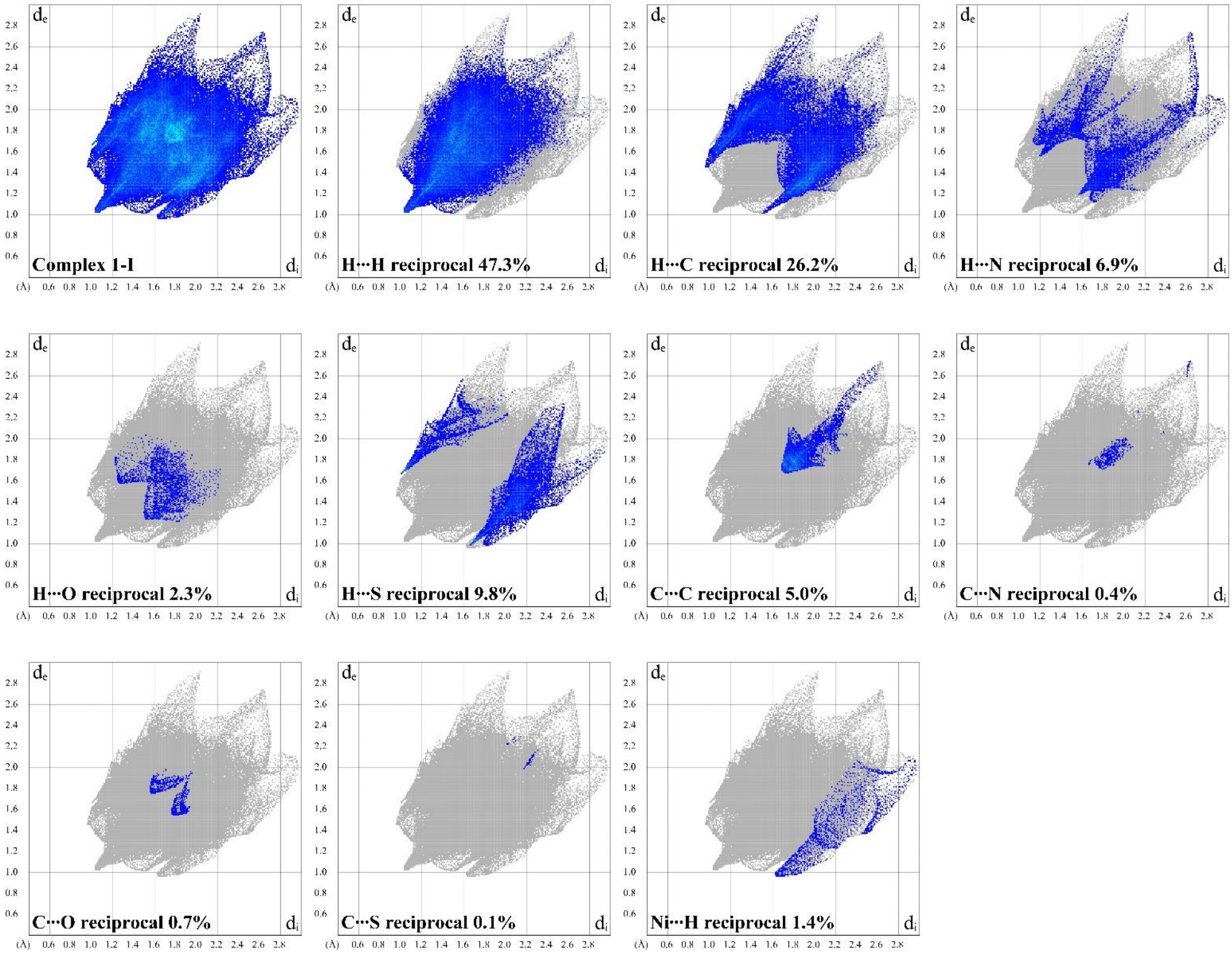

Figure S1. 2D and decomposed 2D fingerprint plots of observed contacts for **1-I**.

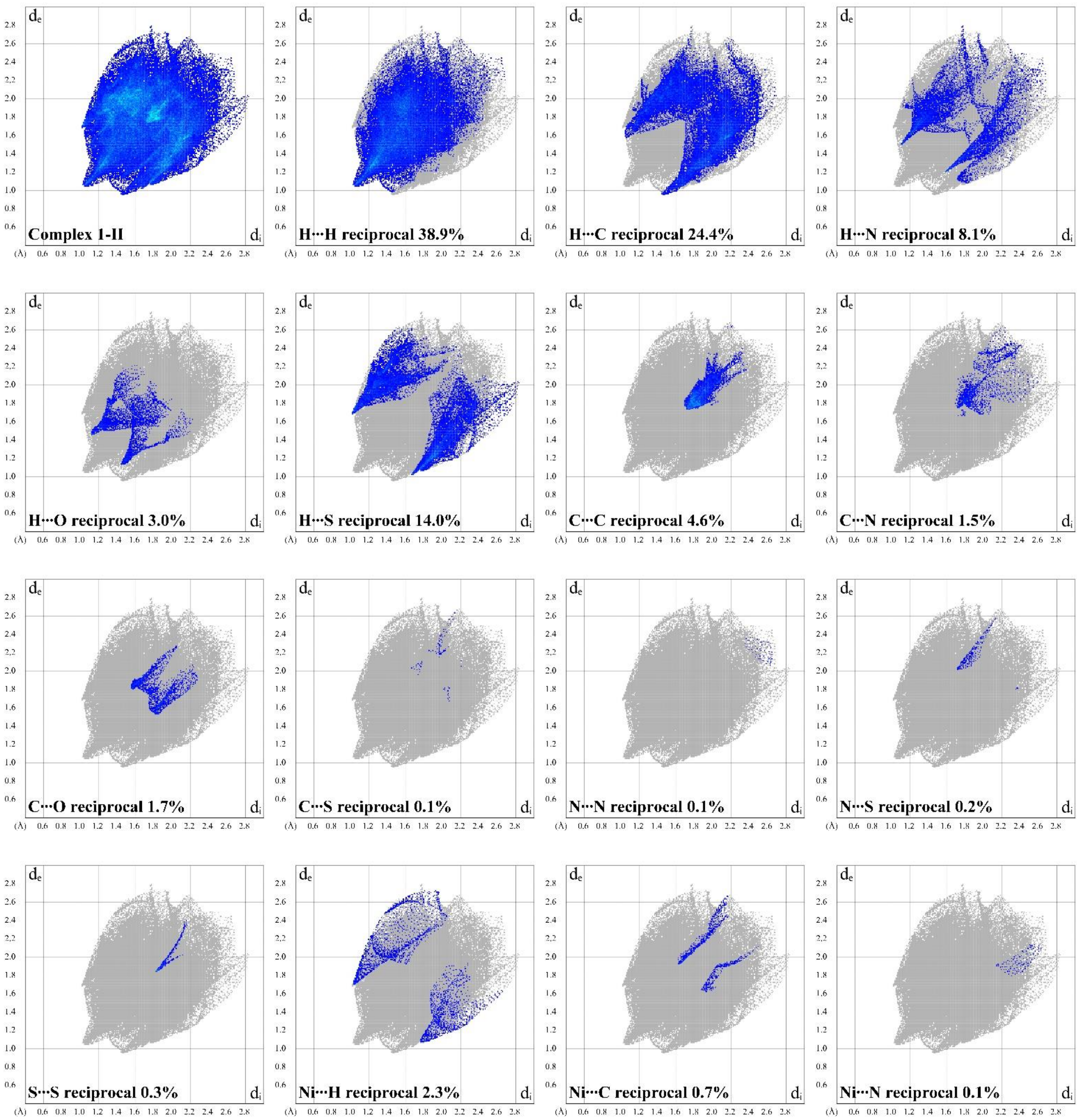

Figure S2. 2D and decomposed 2D fingerprint plots of observed contacts for **1-II**.

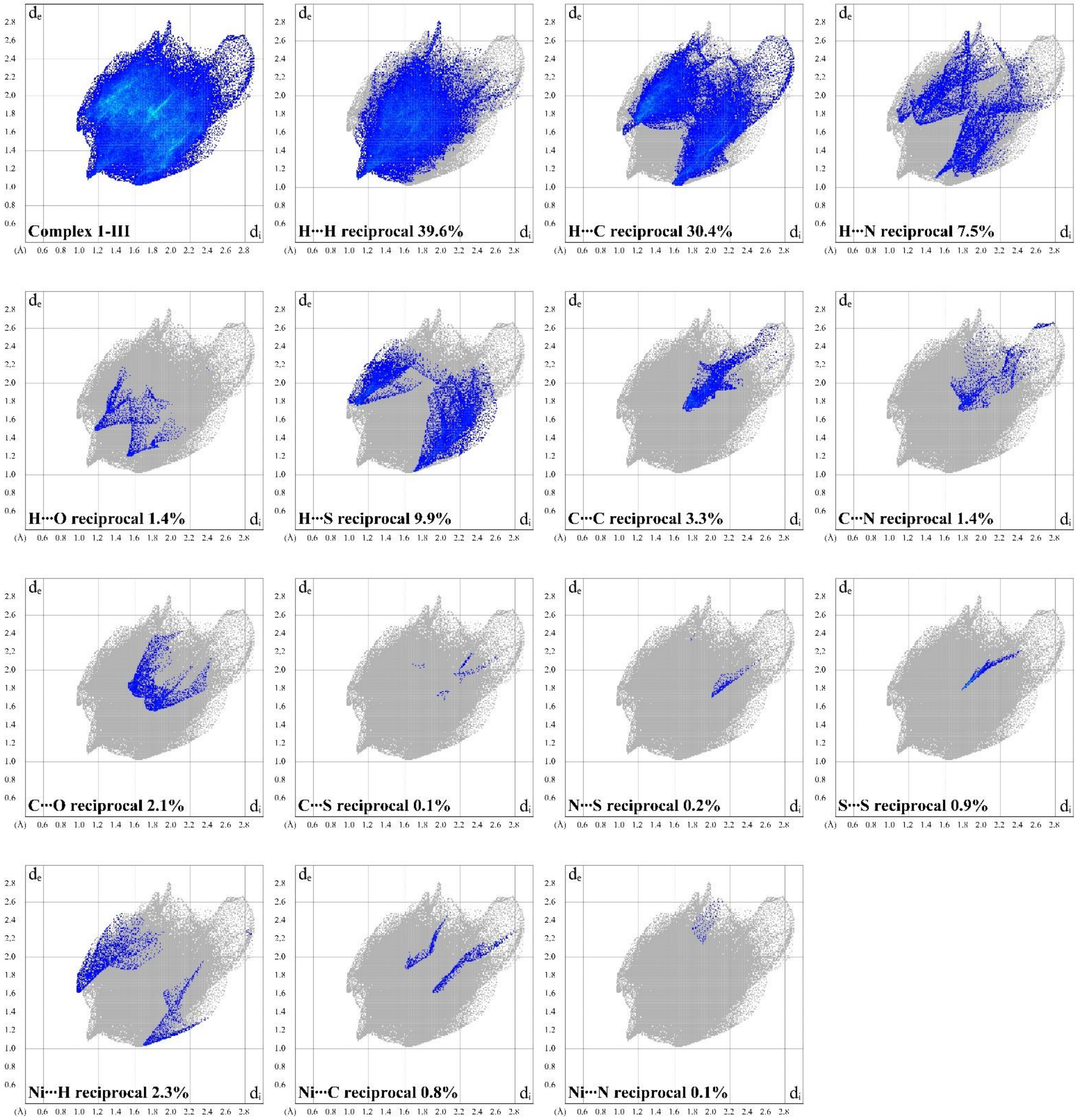

Figure S3. 2D and decomposed 2D fingerprint plots of observed contacts for **1-III**.

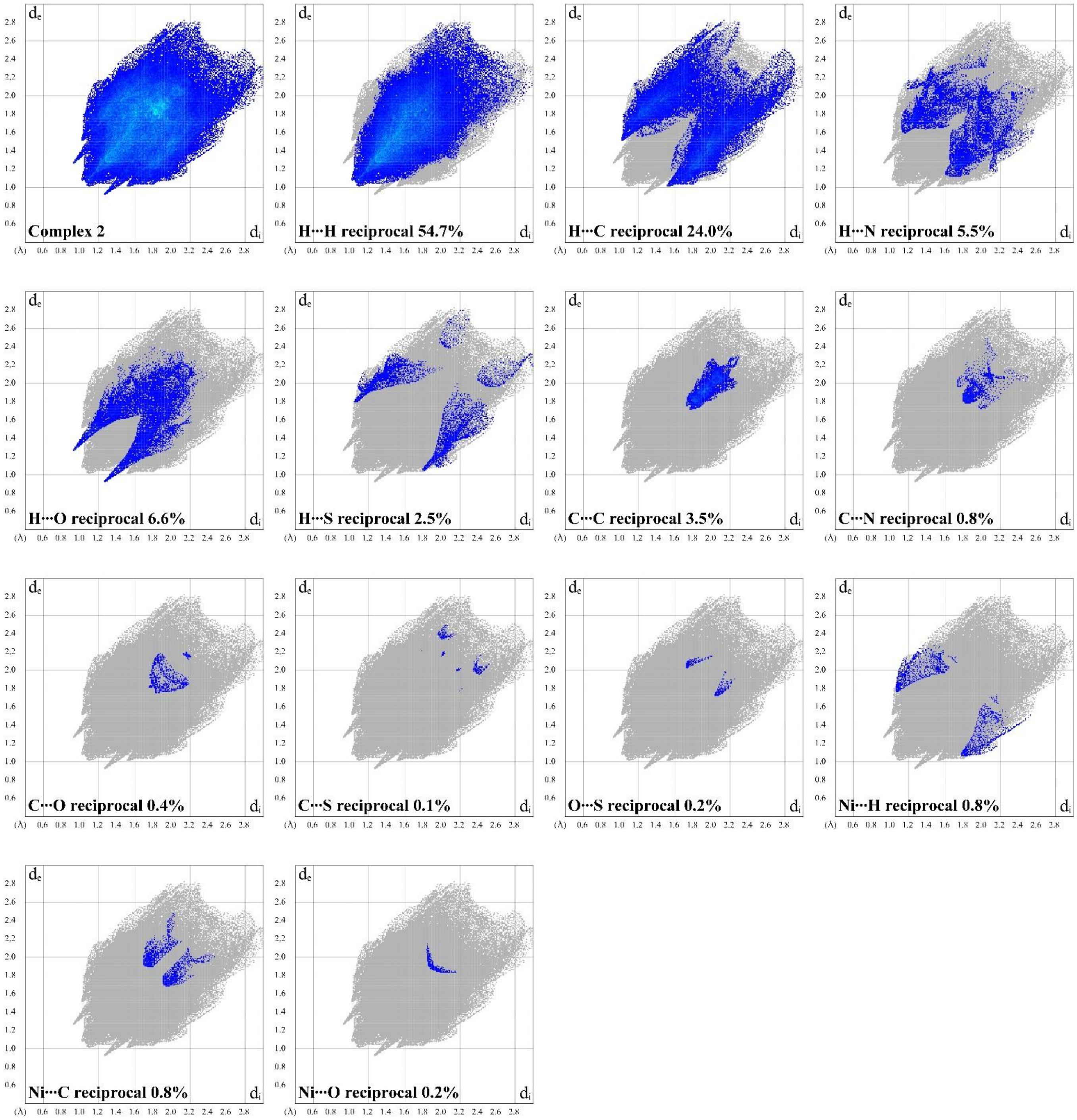

Figure S4. 2D and decomposed 2D fingerprint plots of observed contacts for **2**.
